# Effect of Potassium Permanganate, Ultraviolet Radiation and Titanium Oxide as Ethylene Scavengers on Preservation of Postharvest Quality and Sensory Attributes of Broccoli Stored with Tomatoes

**DOI:** 10.3390/foods12122418

**Published:** 2023-06-20

**Authors:** Ramiro Alonso-Salinas, Santiago López-Miranda, Ana González-Báidez, Antonio José Pérez-López, Luis Noguera-Artiaga, Estrella Núñez-Delicado, Ángel Carbonell-Barrachina, José Ramón Acosta-Motos

**Affiliations:** 1 Plant Biotechnology for Food and Agriculture Group (BioVegA2), Universidad Católica San Antonio de Murcia, Avenida de los Jerónimos 135, Guadalupe, 30107 Murcia, Spain; ralonso@ucam.edu (R.A.-S.); agonzalez@ucam.edu (A.G.-B.); ajperez@ucam.edu (A.J.P.-L.); jracosta@ucam.edu (J.R.A.-M.); 2 Plant Biotechnology, Agriculture and Climate Resilience Group, UCAM-CEBAS-CSIC, Associated Unit to CSIC by CEBAS-CSIC, DP, 30100 Murcia, Spain; 3 Research Group “Food Quality and Safety”, Centro de Investigación e Innovación Agroalimentaria y Agroambiental (CIAGRO-UMH), Miguel Hernández University, Carretera de Beniel, Km 3.2, 03312 Orihuela, Spainangel.carbonell@umh.es (Á.C.-B.); 4 Molecular Recognition and Encapsulation Group (REM), UCAM Universidad Católica de Murcia, Avenida de los Jerónimos 135, Guadalupe, 30107 Murcia, Spain; enunez@ucam.edu

**Keywords:** *Brassica oleracea* var. *italica*, climacteric fruit, ethylene scavengers, *Solanum lycopersicum* L., susceptible vegetable

## Abstract

This study introduces an effective solution to enhance the postharvest preservation of broccoli, a vegetable highly sensitive to ethylene, a hormone produced by climacteric fruits such as tomatoes. The proposed method involves a triple combination of ethylene elimination techniques: potassium permanganate (KMnO_4_) filters combined with ultraviolet radiation (UV-C) and titanium oxide (TiO_2_), along with a continuous airflow to facilitate contact between ethylene and these oxidizing agents. The effectiveness of this approach was evaluated using various analytical techniques, including measurements of weight, soluble solids content, total acidity, maturity index, color, chlorophyll, total phenolic compounds, and sensory analysis conducted by experts. The results demonstrated a significant improvement in the physicochemical quality of postharvest broccoli when treated with the complete system. Notably, broccoli subjected to this innovative method exhibited enhanced organoleptic quality, with heightened flavors and aromas associated with fresh green produce. The implementation of this novel technique holds great potential for the food industry as it reduces postharvest losses, extends the shelf life of broccoli, and ultimately enhances product quality while minimizing waste. The successful development and implementation of this new technique can significantly improve the sustainability of the food industry while ensuring the provision of high-quality food to consumers.

## 1. Introduction

Reducing food waste is a crucial task nowadays due to its economic, social, and environmental impact. It is estimated that worldwide, one-third of all food produced is lost or wasted, which is equivalent to approximately 1.3 billion tons annually [1]. This issue not only poses a problem in terms of wasted resources and loss of profits for farmers and the food industry but also has a negative impact on the environment due to the emission of greenhouse gases and the degradation of ecosystems [2]. One effective way to reduce food waste is through postharvest activities, which occur after harvesting but before distribution and retail sale. Simultaneous storage of crops can be an effective strategy to minimize food waste during the postharvest period.

The simultaneous conservation of broccoli and tomatoes at the same temperatures can have different effects on the agri-food industry due to their distinct optimal storage requirements [3,4,5,6]. Firstly, it is important to consider that broccoli is sensitive to ethylene, while tomatoes are ethylene producers [5,7,8]. When stored together, tomatoes can expedite the ripening process of broccoli due to ethylene release, leading to a reduction in broccoli’s shelf life. Generally, broccoli should be stored at lower temperatures than tomatoes to preserve its freshness and quality for a longer period. At the same temperature, ethylene can cause the broccoli to soften and wilt more rapidly than normal, compromising its shelf life and overall quality. Additionally, direct contact between tomatoes and broccoli can result in surface damage to the broccoli, further impacting its quality. However, the joint conservation of broccoli and tomatoes can yield beneficial effects for the agri-food industry if managed appropriately, such as efficiency in transport, cost reduction, and product availability. For instance, controlled storage techniques can be employed to prolong the freshness of broccoli and minimize the impact of tomato ethylene on its ripening process. Moreover, since tomato and broccoli are two widely consumed crops [9], their combined storage can enhance the efficiency and profitability of distribution and wholesale processes.

Broccoli is an important crop worldwide due to its high nutritional value and growing demand in international markets. According to FAO data [9], over 27 million tons of broccoli were produced worldwide in 2020, with China being the main producer, followed by India, the United States, and Spain. Broccoli is a nutrient-rich cruciferous vegetable that has been associated with a range of health benefits. It is a good source of fiber, vitamin C, vitamin K, folate, iron, and potassium. Additionally, it contains antioxidant and anti-inflammatory compounds such as glucosinolates and carotenoids [10]. Regular consumption of broccoli and other cruciferous vegetables has been shown to be associated with a decreased risk of certain types of cancer, including lung, colon, prostate, and breast cancer. It may also help improve cardiovascular health, reduce inflammation, and enhance cognitive function [11,12]. Furthermore, broccoli is a low-calorie food and can be a healthy option for those looking to lose weight or maintain a healthy weight. Overall, broccoli is highly nutritious and beneficial for health, and its inclusion in a balanced diet is recommended. Tomatoes, on the other hand, are natural producers of ethylene, a plant hormone that is released in a gaseous form and regulates various physiological processes in plants, including ripening and senescence [13,14]. Ethylene production in tomatoes increases during ripening and aging, making them highly ethylene-sensitive fruits [15,16]. Moreover, tomatoes can also impact the ripening and aging of other fruits and vegetables in close proximity, due to ethylene release. For this reason, it is necessary to avoid storing tomatoes together with other fruits and vegetables, especially if they are highly sensitive to ethylene.

There are natural actions that can mitigate the effects of ethylene. Firstly, the amount of ethylene produced by tomatoes can vary depending on their degree of ripeness [5]. Ripe tomatoes produce more ethylene than less ripe ones, so the impact of ethylene on broccoli may be reduced if stored with less ripe tomatoes. Additionally, fresh high-quality broccoli is better able to withstand the effects of tomato ethylene and maintain its quality for a longer period, while lower-quality and less fresh broccoli may be more susceptible to ethylene’s effects. On the other hand, there are several ethylene-removal technologies that can prevent the negative effects of this hormone on sensitive foods such as broccoli [17]. Potassium permanganate [18,19], ultraviolet radiation [20,21], and titanium oxide [22,23] are three different technologies that can be used to eliminate ethylene from the storage environment of fruits and vegetables. Each of these technologies has its own advantages, differences, and mode of action. Potassium permanganate acts by oxidizing ethylene into carbon dioxide and water, while ultraviolet radiation and titanium oxide eliminate ethylene by producing hydroxyl radicals, which react with ethylene to form carbon dioxide and water. Although all three methods aim to remove ethylene, potassium permanganate is more effective in high humidity environments, while ultraviolet radiation and titanium oxide are more effective in low humidity environments. In terms of similarities, the three methods do not require direct contact with the fruits, making them non-invasive and allowing for longer storage periods. Furthermore, all three methods can help delay fruit ripening and extend the shelf life of produce, which is a crucial factor in the food industry.

The aim of this work is to investigate the effect of potassium permanganate, ultraviolet radiation, and titanium oxide on the postharvest conservation of broccoli when it is grown together with tomatoes at the same temperature for a period of 21 days. 

## 2. Materials and Methods

### 2.1. Plant Material

A total of 25 kg of broccoli *Brassica oleracea* var *Italica* were procured from “Hoyamar S.Coop” in Murcia, Spain. The broccoli was harvested using conventional techniques and immediately stored at a temperature of 1 ± 1 °C for 24 h prior to laboratory transportation for subsequent analysis. The classification of the harvested produce based on caliper measurements was conducted by the supplier on the same day of harvest, which took place on 18 May 2022. Furthermore, to ensure homogeneity, a harvest index analysis was performed on a representative sample of 10 randomly selected broccoli florets from the supplied batch, employing the same methodology as the subsequent investigations. The resultant harvest indexes are presented in Table 1. Then, the broccoli was cooled and transported the following day (19 May 2022) to the laboratory. In addition, 25 kg of tomatoes were supplied by “ExpoÁguilas S.Coop” (Murcia, Spain) and were stored alongside the broccoli, except for the control treatment broccoli, to analyse the ethylene produced by this climacteric fruit on a vegetable that is sensitive to this gas, such as broccoli. The choice of tomato for combined storage with broccoli in this study was based on its status as a well-studied climacteric fruit. Additionally, in the production region where the study was conducted (Region of Murcia, Spain), both vegetables share a harvest season for a significant part of the year, and it is common for producing companies to cultivate them together due to the local climatic conditions and production techniques.

### 2.2. Experimental Design

A total of 45 florets of broccoli (25 kg) were randomly distributed into five 150 L (volume) conservation chambers (CCs) (Eurofred Cool Head RCG200, Eurofred S.A., Barcelona, Catalonia, Spain) for the different treatments analyzed. In addition, a total of 85 tomatoes (25 kg) were randomly distributed into 4 (except for the control treatment) 150 L (volume) conservation chambers (CCs) (Eurofred Cool Head RCG200, Eurofred S.A., Barcelona, Catalonia, Spain). 

Following Alonso-Salinas [24], the filters utilized in this study consisted of KMnO_4_ immobilized onto the active centers of zeolite, promoting enhanced interaction between this oxidizing agent and ethylene. The patented filter composition in terms of granulometry and additional adsorbent substances was developed by the Spanish company “Nuevas Tecnologías Agroalimentarias KEEPCOOL” (Molina de Segura, Spain) under patent number 2548787 (2016). To facilitate ethylene removal while preventing the ingress of water or interfering particles, the adsorbing material was enveloped with a semi-permeable paper. This paper allowed the entry of ethylene-rich air while facilitating the egress of purified air free from this phytohormone.

Ethylene filters served as the sole technology for ethylene removal and were installed within an M-CAM 50 device (KEEPCCOL, Molina de Segura, Spain), which functioned as an air-flow-forcing apparatus. This configuration ensured that all air circulating within the conservation chambers (CCs) passed through the ethylene filter. The airflow volume inside the system was 750 L min^−1^, indicating that all the air was recirculated within 12 s, as the chamber had a capacity of 150 L.

**Figure 1:**
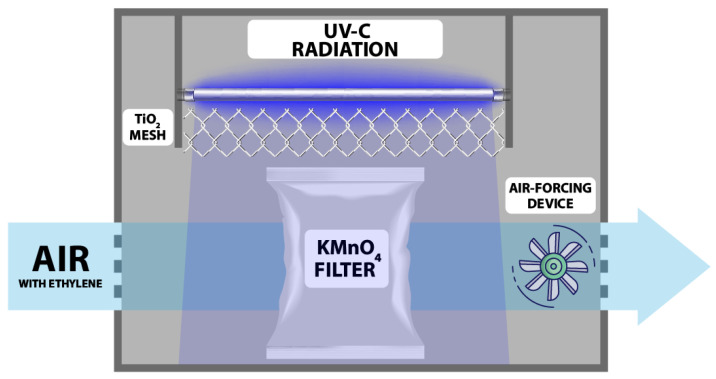
Ethylene scavenger diagram.

Furthermore, this system integrated a photocatalytic ultraviolet light system UV-C (TUV 254 nm, Philips, Amsterdam, The Netherlands) and titanium dioxide TiO_2_ (in mesh format) to support the KMnO_4_ filters in effectively eliminating ethylene. Titanium dioxide is a semiconductor material widely utilized as a photocatalyst for ethylene degradation. Its effectiveness in this role is primarily attributed to its exceptional photochemical reactivity and physical properties, such as high brightness (resulting from its high refractive index) and resistance to discoloration [25]. The mechanism of ethylene elimination through UV light radiation on titanium dioxide is described by Pathak and colleagues [26]. In summary, the reaction is based on the fact that, following irradiation with UV wavelengths (hν) (around 240–380 nm), titanium dioxide generates electrons (e-) that, upon acting on the ambient water, produce highly reactive hydroxyl radicals (·OH). These radicals, in turn, react with organic compounds such as ethylene, resulting in the production of CO_2_ and water [27,28]. To mitigate any potential adverse effects, the application of ultraviolet (UV) light and titanium oxide was targeted specifically at the air exiting the filters rather than the fruit itself. This precautionary measure was taken due to the enclosed nature of the device, which featured only two air inlet and outlet openings. The light beam was directed towards the ethylene component, ensuring that the fruit remained unaffected by the UV light treatment. Figure 1 provides a comprehensive diagram of the system, offering clarity regarding the operational process.

The selection of KMnO_4_, UV-C radiation, and TiO_2_ was based on several factors. Firstly, according to sources in the literature [5,18,25,29], KMnO_4_ was found to be more effective than 1-MCP for ethylene removal. Additionally, KMnO_4_ is more practical for implementation in the food industry due to its affordability compared with palladium. Furthermore, the inclusion of UV light and TiO_2_ was chosen due to their ease of application and their ability to enhance the performance of KMnO_4_.

The treatments were categorized based on ethylene removal, preservation temperature, and relative humidity, as outlined in Table 2.

### 2.3. Physicochemical Variables

All physicochemical analyses were conducted in quintuplicate (*n* = 5) at regular intervals during the entire storage period, specifically on the following days: 0, 7, 15, and 21 (spanning from 19 May 2022 to 8 June 2022). The shelf life of the broccoli was determined to be 21 days, according to the recommended commercial lifetime provided by the supplying company. This time was selected to align with the optimal quality and freshness of the broccoli, ensuring its suitability for commercial purposes. Similar storage durations have also been reported in previous studies [30,31]. 

The concentration of ethylene (C_2_H_4_) was quantified using a gas analyser (Felix Three F-950, Felix Instruments, Camas, WA, USA) and reported in nmol kg^−1^ h^−1^. The gas analyser operated at a measuring flow rate of 1 mL s^−1^, and five measurements were conducted for each analysis and treatment day. To avoid disruption to the internal atmosphere of the chambers, a sealed access point was opened to insert a sonde for ethylene concentration measurements.

The gas analyser had a resolution of 0.1 ppm, and its lower limit of detection was 0.15 ppm. In order to assess potential variations, ethylene measurements were taken 6 h after the beginning of the study on day 0, since the initial ethylene concentration inside the chambers was 0 when the broccoli and tomatoes arrived at the laboratory. This allowed for the observation of any discernible differences in ethylene levels.

The weight measurements were conducted using a precision balance (Navigator balance, Ohaus Europe Gmbh Nänikon, Greifensee, Switzerland), and the results reported in grams. The determination of soluble solid content (SSC), pH, and total acidity (TA) in the fruit samples followed the method adapted from Zhang et al. [32].

To obtain the measurements, 20 g of broccoli was taken and combined with 20 mL of distilled water. The mixture was homogenized for 30 s using an Ultra turrax T25 mixer (LabWare Wilmington, DE, USA). Subsequently, the homogenate was subjected to centrifugation at 3600× *g* for 10 min at 4 °C, employing an Eppendorf centrifuge 5810 (Hamburg, Germany). The resulting supernatant, referred to as “broccoli extract” in the manuscript, was utilized for the determination of SSC, pH, TA, and total phenolic content (TPC).

The soluble solid content (SSC) of the broccoli extract was analyzed using a digital refractometer (Pocket Brix–acidity meter, Atago Tokyo, Japan) at a temperature of 20 °C. The results were expressed as a percentage of sugar equivalent in grams per 100 g (g 100 g^−1^). The pH of the broccoli extract was measured using a pH meter (Testo 206-pH2, Testo, Barcelona, Spain). The determination of total acidity (TA) in the broccoli extract followed the method described by Zang et al. [32] and utilized a Pocket Brix–acidity meter (Atago, Tokyo, Japan). The results of TA were reported as grams per liter (g L^−1^). To assess the maturity index (MI) of the broccoli, the SSC (%) was divided by the TA (%). The MI is a dimensionless parameter that provides insight into the maturity and quality of the broccoli sample.

The total phenolic content (TPC) of the broccoli was determined using a colorimetric method at a wavelength of 765 nm. Folin–Ciocalteu reagent was employed following a modified protocol of Kidron et al. [33]. The Folin–Ciocalteu reaction involved mixing 100 µL of the broccoli extract with 150 µL of Folin–Ciocalteu reagent, 450 µL of 20% Na_2_CO_3_, and 2300 µL of distilled water. After a 2 h dark reaction, the absorbance of the sample was measured against a blank using a spectrophotometer (Shimadzu model UV-1603, Japan). A calibration curve (y = 0.5206x + 0.0899; R^2^ = 0.998) was created using gallic acid as the standard within a range of 25–250 µg mL^−1^. The TPC was expressed as grams of gallic acid equivalent per kilogram of fresh broccoli (g kg^−1^).

The method proposed by Gou et al. [30] was adapted for the analysis of chlorophyll a, chlorophyll b, and total chlorophyll in broccoli samples. A sample weighing 20 g was homogenized using an Ultra Turrax T25 mixer (LabWare, Wilmington, DE, USA) with the addition of 20 g of an acetone–water mixture (80/20 *w*/*w*). The resulting homogenate was centrifuged at 3600× *g* for 5 min. The supernatant was collected after filtration and the absorbance measured at 645 nm and 663 nm using a Shimadzu UV-1603 spectrophotometer (Shimadzu, Japan). Concentrations of chlorophyll a, chlorophyll b, and total chlorophyll were calculated using the following formulas and expressed as mL g^−1^:

(1) $$Chlorophyll\;a=\frac{\left(20.2\times Absorbance645-8.02\times Absorbance663\right)\times V\left(mL\right)}{m\left(g\right)}$$

(2) $$Chlorophyll\;b=\frac{\left(34.7\times Absorbance663-7.12\times Absorbance645)\times V(mL\right)}{m(g)}$$

(3) $$Total\;chlorophyll=\frac{\left(20.2\times Absorbance645+34.7\times Absorbance663\right)\times V(mL)}{m(g)}$$

m = broccoli sample mass expressed in grams.

V = the volume of the extraction solution in millilitres.

The color was determined according to the CIELAB system using a colorimeter (Hunterlab Colorflex EZ, Hunterlab Reston, VA, USA) to measure the color of the florets of five broccolis per treatment and conservation period (0, 7, 15 and 21 days), taking a measurement of CIELAB parameters in two different positions of each fruit and calculating the average. The parameters determined were the coordinates a* (red-green coordinate, red component when positive, green when negative), b* (yellow-blue coordinate, yellow component when positive, blue when negative), and the L parameter, which is a measure of color brightness or lightness referring to the grayscale scale of 0 to 100, where 0 represents absolute black and 100 represents absolute white.

### 2.4. Descriptive Sensory Analysis

The descriptive sensory analysis was carried out by a trained panel comprising 8 highly skilled panellists. The panelists, consisting of 5 females aged between 35 and 56 years, were members of the Food Quality and Safety research group at the Universidad Miguel Hernández de Elche (UMH), Orihuela, Spain. Each panelist possessed extensive experience with fruits and vegetables, with more than 1000 h of training. The methodology employed for the descriptive sensory analysis was previously outlined by Noguera-Artiaga et al. [34]. The scale used ranged from 10 (extremely high intensity) to 0 (no intensity) with 0.5 increments. Prior to the analysis of the samples, 3 training sessions were held in which the lexicon to be used was defined. In these sessions, the panel worked with different broccoli cultivars at day 0 and day 21. From more than 70 descriptors analysed, the final selection was limited to 30, distributed in the visual (green color, yellow color, homogeneity of color, general ID, opening of inflorescences), olfactory (broccoli ID, green vegetable, ripe vegetable, earthy, fermented, sulfurous), gustatory (broccoli ID, green vegetable, ripe vegetable, earthy, fermented, sulfurous, woody, sweet, sour, bitter, astringency, aftertaste), and textural (spicy, hardness, crunchiness, chewiness, juiciness, fibrousness, and residual particles) phases. The samples were presented to the panelists on odor-free disposable plates at room temperature of approximately 22 °C. Each sample was assigned a unique 3-digit code to ensure blind testing. To cleanse their palates between samples, the panelists were provided with mineral water and unsalted crackers. The analyses were conducted in triplicate, with each sample being evaluated three times (*n* = 3).

### 2.5. Statistical Analysis

The descriptive statistics, including the mean and standard error of the mean (SEM), were calculated using StatGraphics Centurion XV software from StatPoint Technologies (Warrenton, VA, USA). The normality of the data was assessed using the Shapiro–Wilk test, and the homogeneity of variance was examined using Bartlett’s test. For the comparison of the 5 treatments across 12 variables on days 7, 15, and 21 of the experiment, a 1-way analysis of variance (ANOVA) was performed. A principal component analysis (PCA) was conducted to identify principal components with eigenvalues greater than or equal to 1.0. Subsequently, a partial least square discriminant analysis was carried out. These analyses explained 82.97% of the variation within the dataset at the end of the 21-day experiment. The sensory analysis data were subjected to an analysis of variance (1-way ANOVA), comparing day 0 with day 21 of the experiment. Tukey’s multiple range test was utilized to compare means and detect significant differences between the treatments (*p*-value < 0.05).

## 3. Results and Discussion

### 3.1. Ethylene

**Figure 2:**
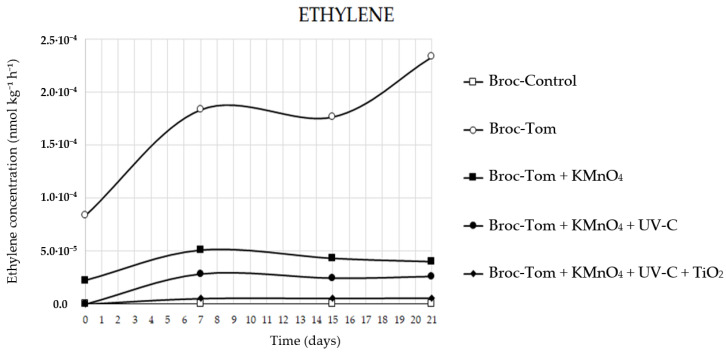
Ethylene concentration (nmol kg^−1^ h^−1^) over the storage period for treatments (Broc-Control, Broc-Tom, Broc-Tom + KMnO_4_, Broc-Tom + KMnO_4_ + UV-C, Broc-Tom + KMnO_4_ + UV-C + TiO_2_).

Regarding ethylene accumulation throughout the assay, the control treatment with only broccoli (Broc-Control) showed little change due to its low ethylene production. The maximum accumulation was observed in the treatment where broccoli and tomato were grown together without any ethylene eliminators (Broc-Tom), indicating that the tomato was responsible for this increase, as a climacteric fruit capable of producing this gas during postharvest after the fruit is separated from the plant. Ethylene decrease was progressive as the number of ethylene eliminators increased (Broc-Tom + KMnO_4_ > Broc-Tom + KMnO_4_ + UV-C > Broc-Tom + KMnO_4_ + UV-C + TiO_2_), with the triple combination treatment accumulating less gas (Broc-Tom + KMnO_4_ + UV-C + TiO_2_) even at levels very close to the control treatment (Broc-Control) (Figure 2). 

Minimizing ethylene concentration during the storage of broccoli is highly desirable to reduce the occurrence of floret damage and internal breakdown. Alonso-Salinas et al. [19] demonstrated a 52% reduction in ethylene concentration in peaches stored at 1 ± 1 °C using an ethylene elimination system incorporating KMnO_4_ + UV-C. Previous studies have examined the individual effectiveness of KMnO_4_ and UV-C as ethylene scavengers [35,36,37]. However, the combination of KMnO_4_ + UV-C employed in this study showed even greater efficacy. Moreover, the present study introduces a novel finding, demonstrating that the triple combination of KMnO_4_ + UV-C + TiO_2_ exhibited an even more significant effect.

In a study by Álvarez-Hernández et al. [38], the application of ethylene scavengers based on KMnO_4_ onto apricots stored at 2 °C led to a reduction close to 100% in ethylene concentration; these results are similar to those obtained in our study when applying the triple combination of KMnO_4_ + UV-C + TiO_2_ to broccoli, but in our study this effect was observed from the beginning of the trial. In recent years, studies on the effect of titanium dioxide–UV on ethylene have focused on its application as a component of films or packaging for fruits. Among them, Fonseca et al. [39] in 2021 evaluated the effectiveness of polyethylene foam nets coated with a photocatalytic nanocomposite composed of gelatine and titanium dioxide to degrade ethylene produced by papayas (*Carica papaya* L.) of the ‘Golden’ variety. The fruits treated with gelatine of titanium dioxide and irradiated with UV light showed a 60% lower ethylene accumulation than the fruit in the control group after four days of storage. De Chiara et al. [40] applied a powder composed of titanium dioxide and silicon dioxide in different proportions for the preservation of tomatoes of the ‘Camone’ variety. In the tomatoes treated with the 80–20 (TiO_2_-SiO_2_) ratio and exposed to UV light, ethylene was completely degraded after 14 days of study. Again, the triple combination of KMnO_4_ + UV-C + TiO_2_ used in our study reduced ethylene concentration in broccoli from the beginning of the trial. 

### 3.2. Physicochemical Variables

Table 3 illustrates the changes in physicochemical variables of broccoli throughout the entire storage period for each treatment. At the end of the experiment (day 21), the treatments Broc-Control and Broc-Tom + KMnO_4_ + UV-C + TiO_2_ demonstrated superior preservation performance and/or longer shelf life compared with the other treatments, and, on the other hand, the Broc-Tomato treatment showed the worst performance. The raw data for weight, pH, SSC, TA, and MI and raw data for color parameters a*, b*, and L are presented in the Supplementary Materials (Supplementary Tables S1 and S2), including the results of the parameters indicated on all days studied (0, 7, 15, and 21). 

After 21 days of study, weight loss was observed in all tested treatments, with the Broc-Tomato treatment experiencing the greatest weight loss, decreasing by up to 39.38% of the initial weight. Among the other treatments, there were no significant differences, but weight loss was lower in the Broc-Control treatment with 43.99% and Broc-Tom + KMnO_4_ + UV-C + TiO_2_ treatment with 35.02% (Table 3). Analysis of how time affected each of the treatments (Supplementary Figure S1) revealed that the pattern of weight loss was the same, with significant differences between day 0 and 7, between day 7 and the other analyzed days, and no differences between day 14 and 21. However, weight loss was faster with the Broc-Tomato treatment. Previous studies on ethylene scavengers also reported a reduction in loss of weight of stored broccoli [41].

After 21 days of testing, a pH increase was observed in all analyzed treatments compared with 0 days but was significantly higher in the Broc-Tomato treatment with 11.42%, compared with the others. Among the other treatments, there were significant differences between Broc-Tom + KMnO_4_ = Broc-Tom + KMnO_4_ + UV-C, with an increase in pH of 8.14% on average, and Broc-Control = Broc-Tom + KMnO_4_ + UV-C + TiO_2_, with an increase in pH of 6.37 on average (Table 3). Analysis of how time affected each of the treatments (Supplementary Figure S1) revealed that the pattern of pH increase was the same, except for the Broc-Tomato treatment which showed significant differences on all days analyzed.

After 21 days was, for both variables in this study, increases and decreases in SSC and AT, respectively, were observed for all treatments studied compared with 0 days of trial. The highest increase in SSC was observed in the Broc-Tomato treatment, with 48.91% compared with day 0. The lowest increase was observed in the Broc-Control treatment, with 22.35%. The treatments with some type of mechanism to eliminate ethylene had intermediate results with average increases of 29.12% (Table 3). In the case of TA, significant decreases were observed only in the Broc-Tomato treatment, with a decrease of 41.94%. The rest of the treatments showed similar responses with average decreases of 23.39% (Table 3). Therefore, an increase in the maturity index, which is calculated as the ratio between total soluble solids and total acidity, is an important indicator of postharvest quality in broccoli, indicating accelerated product ripening that affects its quality and shelf life. The MI also increased over time in this study, with the main differences observed in the Broc-Control (51.76%) and Broc-Tomato (2.57-fold) treatments. Treatments with two (Broc-Tom + KMnO_4_ + UV-C) or three types of mechanisms (Broc-Tom + KMnO_4_ + UV-C + TiO_2_) for ethylene removal responded similarly to the control treatment, with slightly higher average increases of 69.71% (Table 3). Analysis of how time affected each of the treatments (Supplementary Figure S1) revealed that the pattern of SSC increase was the same, except for the Broc-Tomato treatment which showed significant differences on all days analyzed. Regarding decreases in TA, it is noteworthy that there were no differences in any of the treatments studied between day 0 and 7, and that the decreases were not very pronounced on day 14 (Supplementary Figure S1). The increase in the MI showed very similar trends, except in the Broc-Tomato treatment where no significant differences were observed on days 7 and 14 (Supplementary Figure S1).

Phenolic compounds are a class of chemical compounds widely distributed in nature and found in numerous plants, including broccoli. These compounds are important in nutrition due to their antioxidant, anti-inflammatory, antimicrobial, and anticancer properties. In broccoli, phenolic compounds, particularly glucosinolates and flavonoids, are responsible for its health benefits. Glucosinolates are compounds that convert into isothiocyanates when vegetables are cut or chewed, and have been shown to have anticancer and antioxidant properties. Flavonoids are compounds that have antioxidant and anti-inflammatory properties, and have been shown to reduce the risk of cardiovascular diseases and some types of cancer. Additionally, phenolic compounds in broccoli also play an important role in postharvest conservation. Broccoli is a highly perishable vegetable and sensitive to degradation, which can affect its quality and shelf life. It has been demonstrated that phenolic compounds in broccoli have antimicrobial and antioxidant properties, which can help reduce microbial activity and oxidation, thus prolonging the product’s shelf life and improving its quality. After 21 days, there was a decrease in TPC in all treatments, but much greater in the Broc-Tomato treatment with a decrease of 68.06%. The broccoli from Broc-Control and Broc-Tom + KMnO_4_ + UV-C + TiO_2_ treatments with values of approximately 27 g mg^−1^ showed the lowest losses of TPC (25.36%), thus preserving higher nutritional quality (Table 3).

Chlorophyll is a green pigment found in plants and is essential for photosynthesis. In postharvest preservation of broccoli, chlorophyll plays an important role as an indicator of product quality and freshness because its degradation is associated with changes in appearance, flavor, and texture. As chlorophyll degrades, broccoli loses its bright green colour and turns yellowish, indicating a loss of freshness and quality. Therefore, measuring chlorophyll levels in broccoli can be a useful tool for evaluating product quality and shelf life. Specifically, three parameters were measured: chlorophyll a, chlorophyll b, and total chlorophyll. Chlorophyll at the end of the trial had the lowest values in the Broc-Tomato treatment (1.33 mL g^−1^) and the highest values in the Broc-Control (1.81 mL g^−1^) and Broc-Tom + KMnO_4_ + UV-C + TiO_2_ (1.80 mL g^−1^) treatments (Table 3). The trends observed in chlorophyll b and total chlorophyll were the same, with lower values in Broc-Tomato treatment (5.49 and 9.87 mL g^−1^ respectively) and higher but significantly different values in Broc-Control (11.37 and 18.23 mL g^−1^ respectively) and Broc-Tom + KMnO_4_ + UV-C + TiO_2_ (9.08 and 15.41 mL g^−1^ respectively) treatments (Table 3). 

Regarding color, the b* parameter is one of the three color parameters used in the CIELAB scale to measure the color of foods and other objects. The b* parameter describes positive values indicating a yellow component and negative values indicating a blue component. In postharvest conservation of broccoli, the b* parameter is important because it can be used to monitor changes in the colour of the product [42]. As indicated above when broccoli degrades and loses its quality, its bright green colour becomes more yellowish, which is reflected in higher values of the b* parameter. This response in b* was observed in this study with more positive value in Broc-Tomato (24.86) and less positive values in Broc-Control (14.45) and Broc-Tom + KMnO_4_ + UV-C + TiO_2_ (14.61) (Table 3). The evolution over time showed an equal behaviour in all treatments, but with a faster increase in positive values in the Broc-Tomato treatment (Supplementary Figure S2).

Moreover, a* parameter, which describes the red-green colour component of an object (positive values indicating a red component and negative values indicating a green component) has been more negative in Broc-Tomato (−15.26) and less negative values in Broc-Control (−8.17) and Broc-Tom + KMnO_4_ + UV-C + TiO_2_ (−8.48) (Table 3). The evolution over time showed a similar behaviour in all treatments, but with a faster increase in negative values in the Broc-Tomato treatment (Supplementary Figure S2). Finally, the colour parameter L* describes the brightness of an object, with higher values indicating greater brightness and lower values indicating lower brightness. During ethylene-mediated degradation of broccoli during postharvest storage, the decrease in chlorophyll can lead to an increase in brightness and, therefore, in L* values. This is because chlorophyll acts as a dark pigment, so its decrease makes the broccoli appear lighter and brighter. This response in L was observed in this study with more positive values in Broc-Tomato (49.63) and less positive values in Broc-Control (25.63) and Broc-Tom + KMnO_4_ + UV-C + TiO_2_ (26.11) (Table 3). The evolution over time showed equal behavior in all treatments, except in the Broc-Tomato treatment which had a faster increase in positive values (Supplementary Figure S2). 

Other researchers have analyzed the same parameters and ethylene scavengers. For instance, Emadpour et al. [43] observed a weight loss reduction of only 3% during storage with potassium permanganate (without specifying the concentration). Mansourbahmani et al. [18] recorded a 2% weight loss reduction in ‘Valouro’ tomatoes with the application of 20% potassium permanganate mixed with zeolite in a 1:2 ratio, after 35 days of storage. According to Tilahun et al. [44], tissue degradation occurs during ripening, leading to the production of reactive oxygen species (ROS). These substances are eliminated by total phenolic compounds (TPC) and other components that contribute to the fruit’s antioxidant capacity. Mansourbahmani et al. [18] observed that the TPC data after 35 days of treatment by applying 20% potassium permanganate mixed with zeolite in a 1:2 ratio were 35% higher than for untreated tomatoes. Ghosh et al. [45], in 2022, applied photoactivated titanium dioxide and chitosan to create a film for coating ‘Tejaswani’ peppers stored at room temperature (25 °C). The treated peppers exhibited maintained soluble solid content (SSC) along with enhanced color. However, none of these trials adhered to the experimental design planned in this study, which involves growing an ethylene-sensitive vegetable such as broccoli and an ethylene-producing vegetable such as tomato in the same storage chamber. Furthermore, we applied a triple combination of ethylene scavengers, making it challenging to compare our results with those of other researchers.

In summary, in terms of the evolution of the different physicochemical parameters analyzed, the ones that were most affected by the presence of ethylene during storage were MI, SSC, TPC, and total chlorophyll (Table 4).

### 3.3. Sensory Analysis

Table 5 displays the results of the descriptive sensory analysis conducted on broccoli samples. Significant differences were observed in 20 out of the 30 sensory descriptors studied.

Significant differences were found in the color of the samples under study. As expected, the sample stored for 21 days together with the tomato was the one with the least green color intensity and the most yellow color intensity. Initially, the broccoli had an intense green external colour that gradually turned yellow. Following this, the sample was stored for 21 days. In general, the samples in which the influence of the ethylene-regulating treatments was studied preserved the color of the original sample (fresh). Considering the general appearance of the sample, using a descriptor that encompassed color, presence, turgidity (visual), etc., the Broc-Tom + KMnO_4_ + UV-C and the Broc-Tom + KMnO_4_ + UV-C + TiO_2_ treatments were the ones that best preserved the original appearance.

In the case of the smell of the samples, with reference to the characteristic smell of broccoli, none of the treatments studied managed to reproduce the smell of the fresh sample. All the studied treatments obtained much lower intensity values (~2–4, while the fresh sample had an intensity of 10). However, differences were observed in terms of ripe smell. In this case, the Broc-Tom + KMnO_4_ + UV-C + TiO_2_ treatment was the one that, together with the Broc-Control at day 0, presented the lowest intensity, compared with the 21-day treatment, which was the highest. The finding that the treatment effectively blocked the characteristic odor of the ripe product for an extended period highlights its efficacy. The fruit/vegetal aroma significantly influences the overall sensory quality and consumer preference for a fruit. Therefore, preserving the initial aroma of the fruit for a longer duration enhances its market potential [46].

No statistically significant differences were found in the basic flavors of the samples or in the astringency. However, the treatments studied did have an effect in the case of broccoli ID, green vegetable, brown vegetable, earthy, and sulfur flavors. In all cases, globally it could be observed that the ethylene-regulation treatments were able to stop the odors related to the maturity of the samples (mature, earthy, fermented vegetables) and maintain the characteristic odors (broccoli ID, green vegetable, sulfur). The compounds that are most related to the smell and taste of broccoli are isothiocyanates [47]. These give it its characteristic aroma and the longer they are preserved, the more the preservation of their quality can be related.

Upon analyzing the results of the texture analysis, no significant differences were observed in the content of residual particles nor the spiciness of the samples. Differences were apparent in hardness, crunchiness, chewiness, juiciness, and fibrousness. Similar to the case of the odor and taste of the broccoli samples, the ethylene-control treatments also worked to control the texture of the samples. With the use of these systems, all the descriptors related to the quality of the samples, such as hardness or chewiness, were maintained. That is, the sample better preserved its original turgor. In all the texture descriptors evaluated, the sample preserved with tomato was the one that obtained the worst results. 

In view of these results, it can be pointed out that the panel of experts found more appreciable differences in the parameters related to color, firstly, and texture, secondly. In contrast, the sensory attributes related to flavor were not affected for the most part during the storage period under conditions of low ethylene concentration.

### 3.4. Principal Component Analysis

Furthermore, a principal component analysis (PCA) was performed to identify the variables that contributed the most to the overall variability in the experiment and to assess how the different treatments were distinguished from each other. The objective of this analysis was to derive a reduced number of linear combinations from the 12 variables examined (Weight, pH, SSC, TA, MI, TPC, chlorophyll a, chlorophyll b, total chlorophyll, a*, b*, and L*) that captured the most substantial variability present in the data. Two components were extracted since these two components had eigenvalues greater than or equal to 1.0. These components are referred to as principal component 1 (PC1), which explains 74.97% of the experiment’s variability, and principal component 2 (PC2), which explains 8.00% of the experiment’s variability. Together, they accounted for 82.97% of the variability in the original data (Supplementary Table S3 and Figure S3).

Subsequently, the focus shifted to identifying the variables that carried the most weight or exhibited greater importance within each extracted component. In PC1, the variables with the highest weights were determined to be as follows: MI > SSC > TPC > total chlorophyll > L > pH. Similarly, in PC2, the variable with the greatest weight was identified as chlorophyll a (Supplementary Table S4).

**Figure 3:**
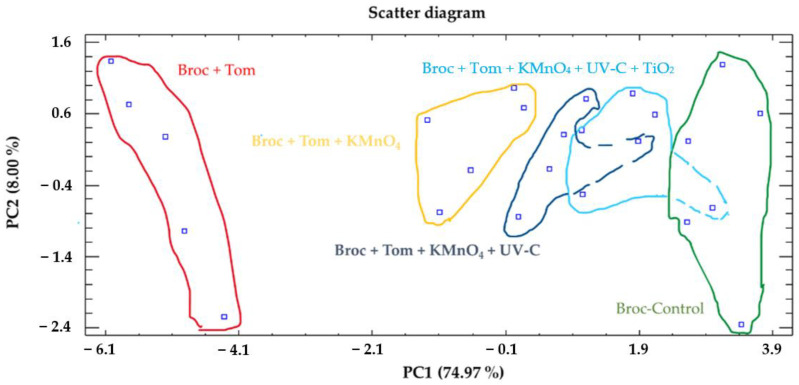
A principal component analysis applied to the different treatments (Broc-Control, Broc-Tomato, Broc-Tom + KMnO_4_, Broc-Tom + KMnO_4_ + UV-C, Broc-Tom + KMnO_4_ + UV-C + TiO_2_). Two principal components (PC1 and PC2) resulted in a model that explained 82.97% of the total variance. Number of replicates *n* = 5.

As mentioned earlier, another objective was to plot the treatments on a scatter diagram (Figure 3) or a biplot (Supplementary Figure S4). These figures were generated using the principal component table where the scores obtained for each component were represented for each treatment (5 per treatment, totaling 25 data points). 

Additionally, the average score for each of the five treatments was included (Supplementary Table S5a). The scatter diagram demonstrated that the treatments were well-separated primarily along the first component (PC1) with a value of F = 116.12 ***, enabling the classification of treatments into five clusters (Supplementary Table S5b): the first cluster consisted of the Broc-Tomato treatment; the second cluster included the Broc-Tom + KMnO_4_ treatment; the third cluster comprised the Broc-Tom + KMnO_4_ + UV-C treatment; the fourth cluster contained the Broc-Tom + KMnO_4_ + UV-C + TiO_2_ treatment; the fifth cluster included the Broc-Control treatment (Supplementary Table S5c). In terms of PC2, the treatments did not form distinct clusters, with a value of F = 0.19 n.s. (Supplementary Table S5d,e).

## 4. Conclusions

In this comprehensive study, we conducted a thorough assessment of ethylene-removal techniques, both individually and in combination, using KMnO_4_, UV-C radiation, and TiO_2_ to preserve the postharvest quality of broccoli. Furthermore, we examined the impact of ethylene produced by tomatoes on ethylene-sensitive broccoli through sensory analysis. The results obtained in this study have significant implications. The application of the aforementioned ethylene-elimination methods demonstrated remarkable efficacy in maintaining exceptionally low levels of this plant hormone, reaching values approaching zero. Notably, when evaluating the condition of the preserved broccoli, the triple combination system (Broc-Tom + KMnO_4_ + UV-C + TiO_2_) demonstrated comparable physicochemical quality to the Broc-Control treatment. This was evident in the increased weight, titratable acidity (TA), total phenolic compounds (TPC), and total chlorophyll content compared with the Broc-Tomato, Broc-Tom + KMnO_4_, and Broc-Tom + KMnO_4_ + UV-C treatments. Additionally, the Broc-Tom + KMnO_4_ + UV-C + TiO_2_ treatment exhibited lower levels of pH, soluble solid content (SSC), and internal browning (MI) compared with other treatments. These findings highlight the efficacy of the ethylene-elimination methods described in this study, particularly the novel triple combination with KMnO_4_, UV-C, and TiO_2_, in effectively delaying the postharvest ripening process of broccoli and extending its shelf life. By employing these innovative techniques, we have made significant strides in preserving the quality of broccoli during postharvest storage. The successful implementation of this approach can have far-reaching implications, including reducing food waste, enhancing marketability, and improving overall sustainability in the food industry.

## Figures and Tables

**Table 1:** Harvest indexes of broccoli and tomato. The means ± standard error of the means (SEM) are shown. *n* = 10.

Parameters	Weight (g)	SSC (%)	TA (%)	Colour
Broccoli	468.71 ± 10	7.64 ± 0.7	1.01 ± 0.05	a*: −4.3 ± 0.5 b*: 6.1 ± 0.8 L*: 24.3 ± 1.9
Tomato	184.22 ± 4.11	5.06 ± 0.12	3.83 ± 0.10	a*: 34.8 ± 3.1 b*: 24.2 ± 2.8 L*: 40.7 ± 4.0
Method	Navigator balance, Ohaus Europe Gmbh (Nänikon, Switzerland).	Pocket Brix–acidity meter, Atago (Tokyo, Japan).	Pocket Brix–acidity meter, Atago (Tokyo, Japan).	Colourpin II, Natural Color System (Stockholm, Sweden).

**Table 2:** Treatment classification according to storage temperature (°C), relative humidity (%) and presence of ethylene scavengers.

Treatments	Broc-Control	Broc-Tomato	Broc-Tom + KMnO_4_	Broc-Tom + KMnO_4_ + UV-C	Broc-Tom + KMnO_4_ + UV-C + TiO_2_
Temperature	1 °C	1 °C	1 °C	1 °C	1 °C
Relative humidity	90%	90%	90%	90%	90%
Ethylene scavenger	None	None	Filter	Filter + UV-C	Filter + UV-C + TiO_2_

**Table 3:** Evolution from day 0 to day 21 of the weight expressed in grams, maturity variables as pH, solid soluble content (SSC) expressed as percentage, total acidity (TA) expressed as g L^−1^, and mature index (MI) as the SSC (%)/TA (%). Total phenol contents (TPC) expressed as mg g^−1^, chlorophyll variables chlorophyll a, chlorophyll b, and total chlorophyll expressed as mL g^−1^, and color parameters a*, b*, and L in broccoli subjected to the different treatments (Broc-Control, Broc-Tom, Broc-Tom + KMnO_4_, Broc-Tom + KMnO_4_ + UV-C, Broc-Tom + KMnO_4_ + UV-C + TiO_2_).

Treatments	Weight (%)		pH		SSC (%)	
	Day 0	Day 21	Day 0	Day 21	Day 0	Day 21
Broc-Control	100	56.01 ± 3.08 a	6.39 ± 0.03	6.78 ± 0.04 c	7.83 ± 0.33	9.58 ± 0.07 c
Broc-Tomato		39.38 ± 1.41 c		7.12 ± 0.04 a		11.66 ± 0.10 a
Broc-Tom + KMnO_4_		45.56 ± 1.49 b		6.95 ± 0.03 b		10.33 ± 0.07 b
Broc-Tom + KMnO_4_ + UV-C		51.20 ± 2.38 a		6.87 ± 0.01 b		9.99 ± 0.09 b
Broc-Tom + KMnO_4_ + UV-C + TiO_2_		64.98 ± 3.50 a		6.82 ± 0.02 c		10.01 ± 0.10 b
		F = 6.21 **		F = 17.96 ***		F = 86.33 **
Treatments	TA		MI		TPC	
	Day 0	Day 21	Day 0	Day 21	Day 0	Day 21
Broc-Control	0.93 ± 0.05	0.75 ± 0.02 a	1.70 ± 0.09	2.58 ± 0.06 c	36.41 ± 1.24	27.10 ± 0.29 a
Broc-Tomato		0.54 ± 0.01 b		4.37 ± 0.08 a		11.63 ± 1.02 c
Broc-Tom + KMnO_4_		0.69 ± 0.02 a		3.01 ± 0.10 b		20.11 ± 1.57 b
Broc-Tom + KMnO_4_ + UV-C		0.71 ± 0.03 a		2.86 ± 0.14 bc		24.47 ± 0.78 ab
Broc-Tom + KMnO_4_ + UV-C + TiO_2_		0.70 ± 0.02 a		2.90 ± 0.10 bc		27.25 ± 0.60 a
		F = 12.84 ***		F = 49.31 ***		F = 46.96 ***
Treatments	Chlorophyll a		Chlorophyll b		Total Chlorophyll	
	Day 0	Day 21	Day 0	Day 21	Day 0	Day 21
Broc-Control	2.19 ± 0.26	1.81 ± 0.24 a	10.41 ± 1.13	11.37 ± 0.52 a	18.62 ± 1.26	18.23 ± 0.38 a
Broc-Tomato		1.33 ± 0.21 b		5.49 ± 0.68 d		9.87 ± 0.62 d
Broc-Tom + KMnO_4_		1.60 ± 0.13 ab		7.22 ± 0.23 c		12.68 ± 0.31 c
Broc-Tom + KMnO_4_ + UV-C		1.62 ± 0.09 ab		8.13 ± 0.28 bc		13.91 ± 0.36 bc
Broc-Tom + KMnO_4_ + UV-C + TiO_2_		1.80 ± 0.12 a		9.08 ± 0.29 b		15.41 ± 0.22 b
		F = 4.84 *		F = 35.65 ***		F = 60.06 ***
Treatments	Colour a*		Colour b*		Colour L	
	Day 0	Day 21	Day 0	Day 21	Day 0	Day 21
Broc-Control	−4.52 ± 0.23	−8.17 ± 0.39 a	6.26 ± 0.30	14.45 ± 0.52 b	25.69 ± 0.68	25.63 ± 1.41 b
Broc-Tomato		−15.26 ± 1.56 c		24.86 ± 1.36 a		49.63 ± 2.96 a
Broc-Tom + KMnO_4_		−12.69 ± 1.02 bc		17.18 ± 0.69 b		30.33 ± 1.22 b
Broc-Tom + KMnO_4_ + UV-C		−10.80 ± 0.47 ab		15.04 ± 0.94 b		28.95 ± 0.86 b
Broc-Tom + KMnO_4_ + UV-C + TiO_2_		−8.48 ± 0.27 a		14.61 ± 1.19 b		26.11 ± 0.92 b
		F = 11.28 ***		F = 19.83 ***		F = 36.08 ***

The means ± standard error of the means (SEM) are shown. Different letters for each treatment represent statistically significant differences according to Tukey’s test, *n* = 5 per treatment and day. *, **, ***, significant at *p* < 0.05, 0.01, and 0.001, respectively.

**Table 4:** Summary table of the effect of ethylene on physico-chemical parameters. Mature index (MI); solid soluble content (SSC); Total phenol contents (TPC); Total acidity (TA); colour parameters as a*, b*, and L. F values express sensibility to ethylene. Higher values are correlated with higher sensitivity.

Parameters	F
MI	320.83 ***
SSC	306.50 ***
TPC	215.00 ***
Total chlorophyll	132.27 ***
TA	70.90 ***
Chlorophyll b	63.29 ***
L	53.74 ***
b*	51.16 ***
pH	34.46 ***
Weight	20.29 **
a*	19.43 **
Chlorophyll a	2.31 n.s.

**, ***, significant at *p* < 0.01, and 0.001, respectively. n.s., no significant.

**Table 5:** Descriptive sensory analysis of broccoli.

		Day 0	Day 21				
Sensory Descriptor	ANOVA ^‡^	Broc-Control	Broc-Control	Broc-Tomato	Broc-Tom + KMnO_4_	Broc-Tom + KMnO_4_ + UV-C	Broc-Tom + KMnO_4_ + UV-C + TiO_2_
COLOR							
Green color	***	9.5 b	5.5 a	2.5 c	8.5 b	8.5 b	8.5 b
Yellow color	***	0.5 d	1.5 c	6.0 a	2.5 b	2.5 b	1.5 c
Colour homogeneity	***	8.6 a	7.3 c	5.0 d	7.5 bc	7.8 abc	8.5 ab
External broccoli ID	***	9.0 a	2.7 c	3.2 c	6.7 b	8.0 ab	8.7 a
Inflorescences (closed)	***	9.3 ab	10.0 a	6.0 d	7.5 c	8.7 b	8.8 b
ODOR							
Broccoli ID	***	10.0 a	4.3 b	1.8 b	2.3 b	2.8 b	2.7 b
Green vegetable	***	9.5 a	2.6 b	1.0 b	2.1 b	2.0 b	2.0 b
Ripe vegetable	***	0.3 d	7.3 a	5.3 b	5.0 b	5.8 b	3.7 c
Earthy	NS	1.8	5.0	3.7	2.8	3.2	3.3
Fermented	NS	0.0	1.3	2.0	0.8	0.7	1.0
Sulfurous	**	8.0 a	4.3 ab	3.3 b	3.3 b	4.0 b	3.3 b
FLAVOR							
Broccoli ID	***	9.3 a	4.6 cd	3.0 d	6.5 bc	8.0 ab	5.8 c
Green vegetable	***	9.5 a	2.3 c	1.5 c	5.1 b	6.3 b	4.7 b
Ripe vegetable	***	0.1 b	7.6 a	6.0 a	4.8 ab	3.7 ab	4.3 ab
Earthy	*	0.7 b	3.6 a	3.5 a	2.0 b	1.3 b	1.5 b
Fermented	NS	0.0	0.8	1.5	0.0	0.3	1.0
Sulfurous	***	8.0 a	4.5 cd	2.7 d	5.8 bc	6.5 ab	5.5 bc
Woody	NS	0.0	0.8	1.0	0.0	0.5	0.0
Sweet	NS	3.0	4.0	3.5	5.3	4.3	4.3
Sour	NS	2.2	1.8	1.3	1.0	1.0	1.3
Bitter	NS	4.0	3.5	4.1	2.5	3.0	3.5
Astringency	NS	1.0	0.8	1.1	0.6	1.0	1.1
Aftertaste	**	8.3 a	4.8 c	4 c	6.5 b	5.8 b	6.3 b
TEXTURE							
Spicy	NS	2.7	1.8	1.3	2.1	2.5	2.7
Hardness	**	9.0 a	9.0 a	6.7 b	7.5 ab	8.5 a	9.5 a
Crunchiness	***	7.1 a	2.7 c	2.5 c	4.3 bc	6.1 ab	4.7 bc
Chewiness	***	8.0 b	9.3 a	9.7 a	8.1 b	8.6 ab	9.3 a
Juiciness	***	3.0 a	0.7 c	0.8 bc	2.0 abc	2.7 ab	1.7 abc
Residual particles	NS	5.0	6.0	6.5	6.3	5.5	6.5
Fibrousness	**	0.3 c	2.3 b	4.3 a	1.8 b	2.0 b	2.3 b

^‡^ NS = not significant at *p* > 0.05; *, **, ***. significant at *p* < 0.05, 0.01, and 0.001, respectively. Values (mean of three replications) followed by the same letter within the same sensory descriptor are not significantly different (*p* > 0.05) according to Tukey’s least significant difference test.

## Data Availability

All related data and methods are presented in this paper. Additional inquiries should be addressed to the corresponding autor.

## Supplementary Materials

The following supporting information can be downloaded at: https://www.mdpi.com/article/10.3390/foods12122418/s1, Supplementary Figure S1: Evolution during storage time of weight, pH, SSC, TA, and MI expressed in broccoli subjected to different treatments (Broc-Control, Broc-Tomato, Broc-Tom + KMnO_4_, Broc-Tom + KMnO_4_ + UV-C and Broc-Tom + KMnO_4_ + UV-C + TiO_2_); Supplementary Figure S2: Evolution during storage time of the color parameters in broccoli subjected to different treatments (Broc-Control, Broc-Tomato, Broc-Tom + KMnO_4_, Broc-Tom + KMnO_4_ + UV-C and Broc-Tom + KMnO_4_ + UV-C + TiO_2_); Supplementary Figure S3: Sedimentation graph; Supplementary Figure S4: Graphical representation of the principal components marked with lines for each variable and with points for each score; Supplementary Table S1: Evolution during storage time of the physical variables in broccoli florets subjected to the different treatments: (Broc-Control, Broc-Tomato, Broc-Tom + KMnO_4_, Broc-Tom + KMnO_4_ + UV-C and Broc-Tom + KMnO_4_ + UV-C + TiO_2_); Supplementary Table S2: Evolution during storage time of the biochemical variables in broccoli florets subjected to different treatments: (Broc-Control, Broc-Tomato, Broc-Tom + KMnO_4_, Broc-Tom + KMnO_4_ + UV-C and Broc-Tom + KMnO_4_ + UV-C + TiO_2_); Supplementary Table S3: Principal component analysis; Supplementary Table S4: Table of component weights; Supplementary Table S5a: This table shows the scores of the principal components; Supplementary Table S5b: ANOVA table for the component 1 scores according to the treatments; Supplementary Table S5c: Multiple comparisons test for the component 1 scores by treatment using Tukey’s HSD method; Supplementary Table S5d: ANOVA table for the component 2 scores according to the treatments; Supplementary Table S5e: Multiple comparisons test for the component 2 scores by treatment using Tukey’s HSD method.
