# Supplementary material for: Effect of Potassium Permanganate, Ultraviolet Radiation and Titanium Oxide as Ethylene Scavengers on Preservation of Postharvest Quality and Sensory Attributes of Broccoli Stored with Tomatoes
Source: Foods. 2023 Jun 20;12(12):2418. doi: 10.3390/foods12122418 (PMC10297595; doi:10.3390/foods12122418)
Supplement: Supplementary file 1 [file foods-12-02418-s001.zip › foods-2431464-supplementary.pdf]

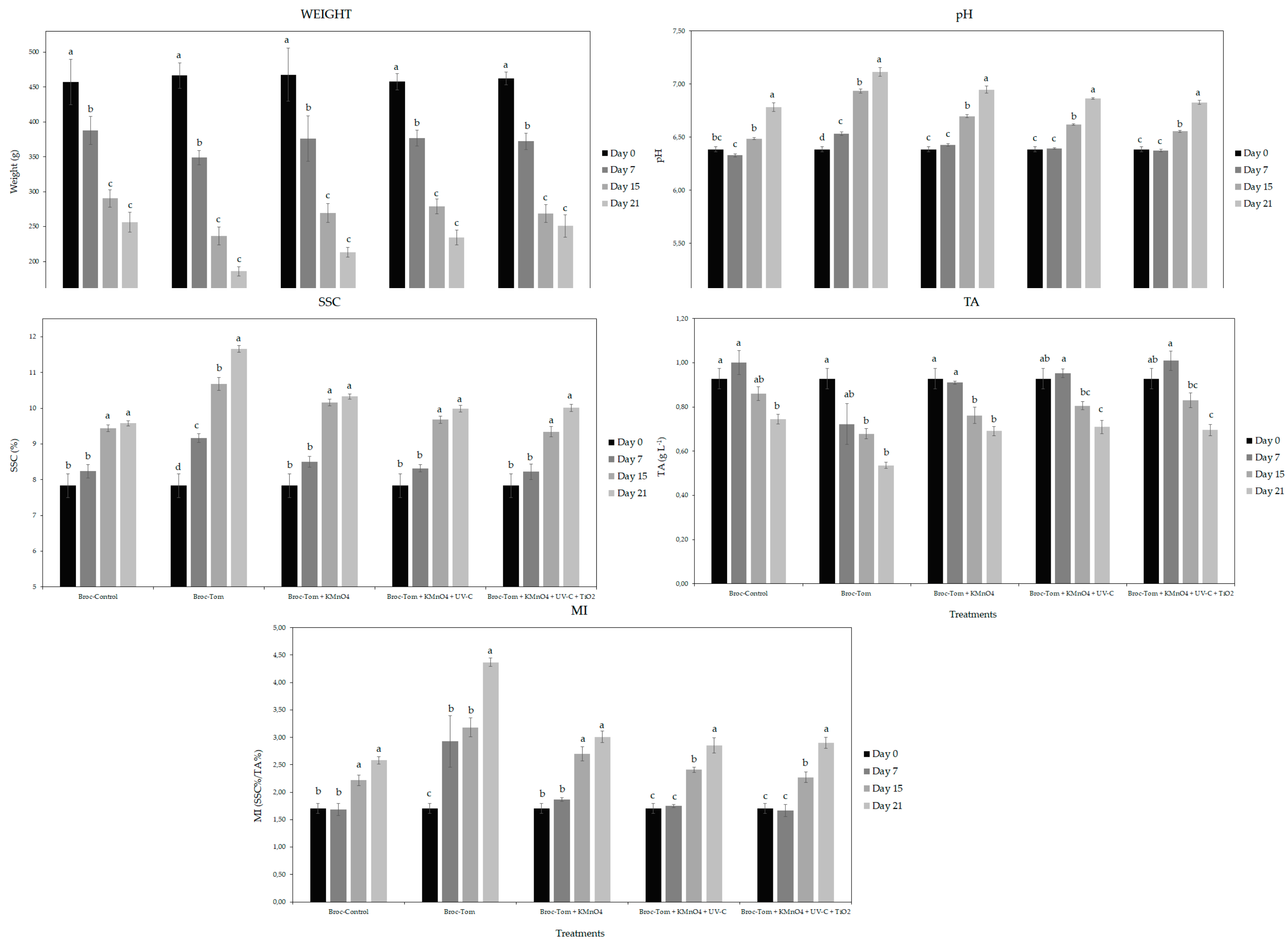

Supplementary Figure S1. Evolution during storage time of the broccoli's weight expressed in grams. pH. SSC expressed as percentage. TA expressed as g L^{-1} and MI expressed as the SSC (%)/TA (%) ratio subjected to different treatments (Broc-Control. Broc-Tom. Broc-Tom + $KMnO_4$. Broc-Tomato + $KMnO_4$ + UV-C. Broc-Tom + $KMnO_4$ + UV-C + TiO_2). Different letters for each treatment represent statistically significant differences according to Tukey's test. n = 5. with the aim to see the evolution of each parameter on every treatment.

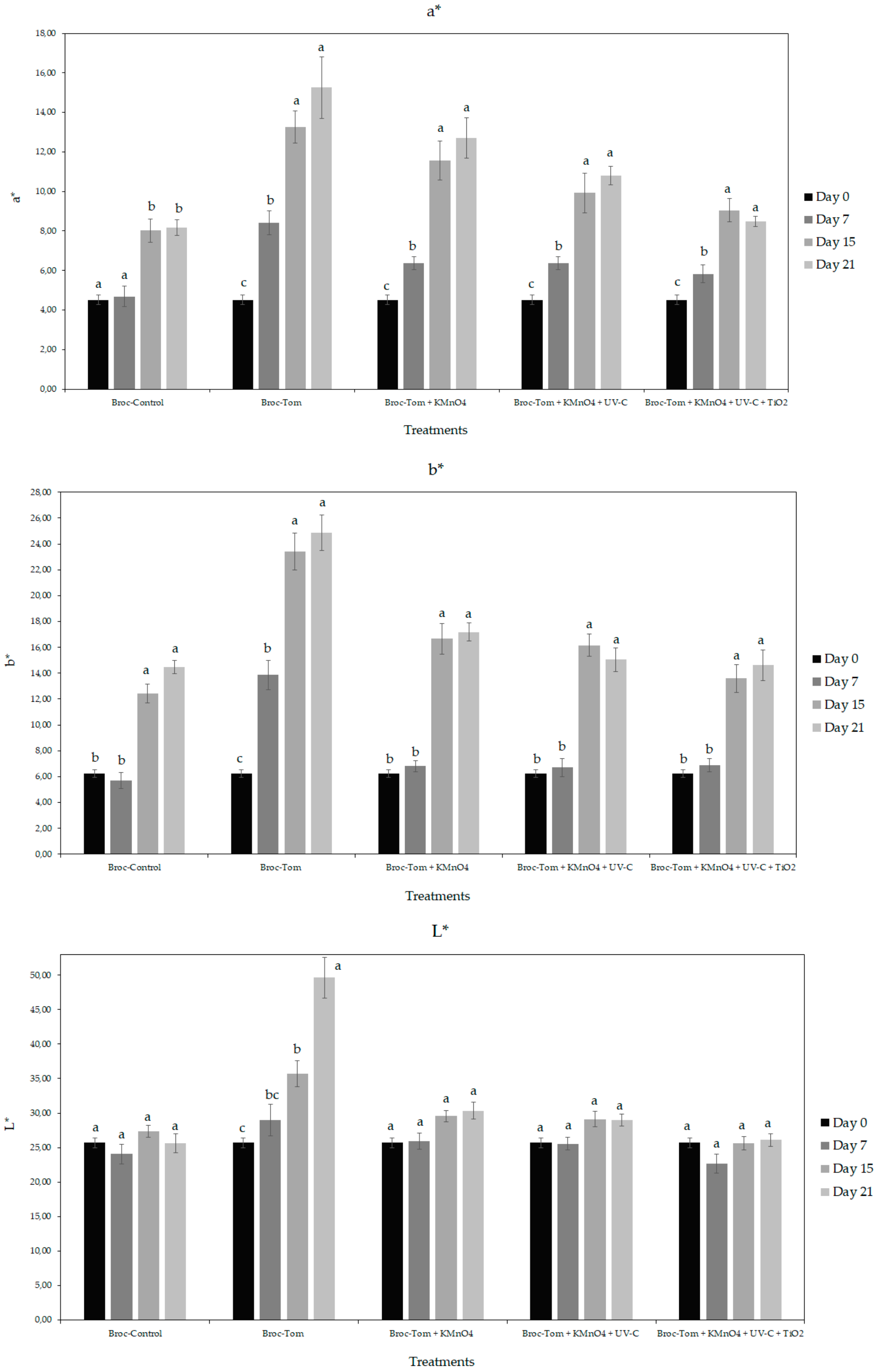

Supplementary Figure S2. Evolution during storage time of the colour parameters in broccoli subjected to different treatments (Broc-Control. Broc-Tomato. Broc-Tom + $KMnO_4$. Broc-Tom + $KMnO_4$ + UV-C. Broc-Tom + $KMnO_4$ + UV-C + TiO_2). The parameters measured were a* (a); b* (b) and L* (c). Different letters for each treatment represent statistically significant differences according to Tukey's test. n = 5. with the aim to see the evolution of each parameter on every treatment

Supplementary Table S1. Evolution from day 0 to day 21 of the weight expressed in grams and maturity variables as solid soluble content (SSC) expressed as percentage; pH; Total acidity (TA) expressed as g L^{-1} and mature index (MI) as the SSC (%)/TA (%) ratio in broccoli subjected to the different treatments (Broc-Control. Broc-Tomato. Broc-Tom + $KMnO_4$. Broc-Tom + $KMnO_4$ + UV-C. Broc-Tom + $KMnO_4$ + UV-C + TiO_2). The means ± standard error of the means (SEM) are shown. Different letters for each treatment represent statistically significant differences according to Tukey's test. n = 5 per treatment and day.

Storage days	Treatment	Weight (g)	SSC (%)	pH	TA (g L^{-1})	MI (SSC/TA)
0	All	449.47 ± 11.44	7.83 ± 0.33	6.39 ± 0.03	0.93 ± 0.05	1.70 ± 0.09
7	Broc-Control	387.91 ± 20.13 a	8.24 ± 0.19 b	6.33 ± 0.01 d	1.00 ± 0.05 a	1.69 ± 0.11 b
	Broc-Tomato	348.83 ± 10.37 a	9.16 ± 0.12 a	6.53 ± 0.02 a	0.72 ± 0.09 b	2.93 ± 0.47 a
	Broc-Tom + $KMnO_4$	376.07 ± 32.78 a	8.50 ± 0.15 ab	6.43 ± 0.01 b	0.91 ± 0.01 ab	1.87 ± 0.03 b
	Broc-Tom + $KMnO_4$ + UV-C	376.67 ± 11.51 a	8.32 ± 0.10 b	6.39 ± 0.01 bc	0.95 ± 0.02 a	1.75 ± 0.03 b
	Broc-Tom + $KMnO_4$ + UV-C + TiO_2	404.06 ± 11.69 a	8.22 ± 0.22 b	6.37 ± 0.01 cd	1.01 ± 0.04 a	1.66 ± 0.11 b
15	Broc-Control	290.48 ± 12.30 a	9.44 ± 0.10 c	6.49 ± 0.01 e	0.86 ± 0.03 a	2.22 ± 0.10 b
	Broc-Tomato	236.52 ± 13.13 b	10.68 ± 0.18 a	6.94 ± 0.02 a	0.68 ± 0.02 b	3.18 ± 0.17 a
	Broc-Tom + $KMnO_4$	269.45 ± 13.18 ab	10.16 ± 0.09 ab	6.70 ± 0.01 b	0.76 ± 0.04 ab	2.70 ± 0.13 ab
	Broc-Tom + $KMnO_4$ + UV-C	278.75 ± 10.49 a	9.68 ± 0.10 bc	6.62 ± 0.01 c	0.81 ± 0.02 a	2.41 ± 0.05 b
	Broc-Tom + $KMnO_4$ + UV-C + TiO_2	326.17 ± 12.58 a	9.34 ± 0.15 c	6.55 ± 0.01 d	0.83 ± 0.03 a	2.27 ± 0.09 b
21	Broc-Control	256.06 ± 14.09 a	9.58 ± 0.07 c	6.78 ± 0.04 c	0.75 ± 0.04 a	2.58 ± 0.06 c
	Broc-Tomato	186.04 ± 6.59 b	11.66 ± 0.10 a	7.12 ± 0.04 a	0.54 ± 0.04 b	4.37 ± 0.08 a
	Broc-Tom + $KMnO_4$	213.11 ± 6.99 ab	10.33 ± 0.07 b	6.95 ± 0.03 b	0.69 ± 0.03 a	3.01 ± 0.10 b
	Broc-Tom + $KMnO_4$ + UV-C	234.27 ± 10.89 ab	9.99 ± 0.09 b	6.87 ± 0.01 bc	0.71 ± 0.01 a	2.86 ± 0.14 bc
	Broc-Tom + $KMnO_4$ + UV-C + TiO_2	300.35 ± 16.18 a	10.01 ± 0.10 b	6.83 ± 0.02 bc	0.70 ± 0.02 a	2.90 ± 0.10 bc

Supplementary Table S2. Evolution from day 0 to day 21 of the colour variables in broccoli subjected to the different treatments (Broc-Control. Broc-Tomato. Broc-Tom + $KMnO_4$. Broc-Tom + $KmnO_4$ + UV-C. Broc-Tom + $KmnO_4$ + UV-C + TiO_2). The parameters measured were a*; b* and L*. The means ± standard error of the means (SEM) are shown. Different letters for each treatment represent statistically significant differences according to Tukey's test. n = 5 per treatment and day.

Storage days	Treatment	a*	b*	L*
0	All	-4.52 ± 0.23	6.26 ± 0.30	25.69 ± 0.68
7	Broc-Control	-4.69 ± 0.51 a	5.69 ± 0.60 b	24.06 ± 1.46 a
	Broc-Tomato	-8.42 ± 0.61 b	13.87 ± 1.12 a	28.99 ± 2.26 a
	Broc-Tom + $KmnO_4$	-6.37 ± 0.32 a	6.80 ± 0.44 b	25.92 ± 1.17 a
	Broc-Tom + $KmnO_4$ + UV-C	-6.37 ± 0.34 a	6.71 ± 0.69 b	25.52 ± 0.92 a
	Broc-Tom + $KmnO_4$ + UV-C + TiO_2	-5.83 ± 0.45 a	6.88 ± 0.53 b	22.71 ± 1.38 a
15	Broc-Control	-8.03 ± 0.59 a	12.45 ± 0.72 b	27.37 ± 0.86 b
	Broc-Tomato	-13.27 ± 0.81 c	23.43 ± 1.43 a	35.73 ± 1.90 a
	Broc-Tom + $KmnO_4$	-11.57 ± 0.98 bc	16.66 ± 1.17 b	29.55 ± 0.77 b
	Broc-Tom + $KmnO_4$ + UV-C	-9.93 ± 1.00 abc	16.17 ± 0.84 b	29.13 ± 1.16 b
	Broc-Tom + $KmnO_4$ + UV-C + TiO_2	-9.05 ± 0.59 ab	13.59 ± 1.05 b	25.63 ± 0.94 b
21	Broc-Control	-8.17 ± 0.39 a	14.45 ± 0.52 b	25.63 ± 1.41 a
	Broc-Tomato	-15.26 ± 1.56 c	24.86 ± 1.36 a	49.63 ± 2.96 b
	Broc-Tom + $KmnO_4$	-12.69 ± 1.02 bc	17.18 ± 0.69 b	30.33 ± 1.22 a
	Broc-Tom + $KmnO_4$ + UV-C	-10.80 ± 0.47 ab	15.04 ± 0.94 b	28.95 ± 0.86 a
	Broc-Tom + $KmnO_4$ + UV-C + TiO_2	-8.48 ± 0.27 a	14.61 ± 1.19 b	26.11 ± 0.92 a

Supplementary Table S3. Principal Component Analysis

Component	*Eugenvalue*	*Percentage of Variance*	*Percentage Cumulative*
1	8.99666	74.972	74.972
2	0.959885	7.999	82.971
3	0.584659	4.872	87.843
4	0.449655	3.747	91.590
5	0.392415	3.270	94.861
6	0.22385	1.865	96.726
7	0.15998	1.333	98.059
8	0.137825	1.149	99.208
9	0.0543274	0.453	99.660
10	0.0308153	0.257	99.917
11	0.00594387	0.050	99.967
12	0.00398482	0.033	100.000

Supplementary Figure S3. Sedimentation graph

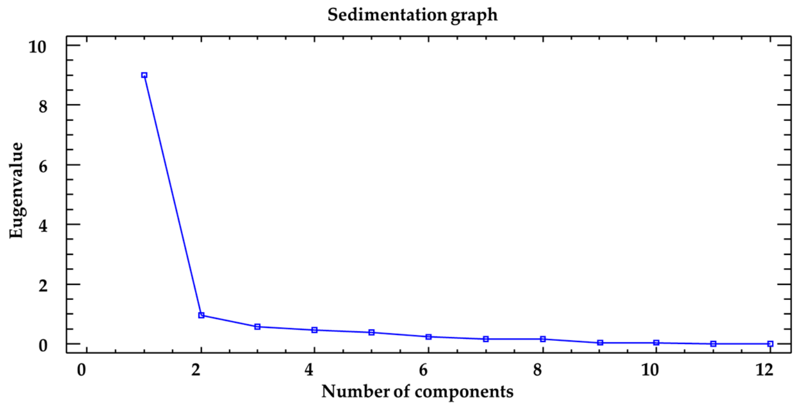

Supplementary Table S4. Table of Component Weights

	Component	*Component*
	1	2
Weight	0.270102	-0.0855683
pH	**-0.304243**	0.153234
SSC	**-0.312978**	-0.139678
TA	0.296331	0.0863789
MI	**-0.317859**	-0.0894007
TPC	**0.312048**	0.0834933
chlorophyll a	0.142592	**0.890264**
chlorophyll b	0.291496	-0.327893
Total chlorophyll	**0.30604**	-0.138452
a*	0.262856	-0.0363338
b*	-0.299334	0.038533
L	**-0.304382**	0.0721456

Supplementary Figure S4. Graphical representation of the principal components marking with lines for each variable and wit points for each score

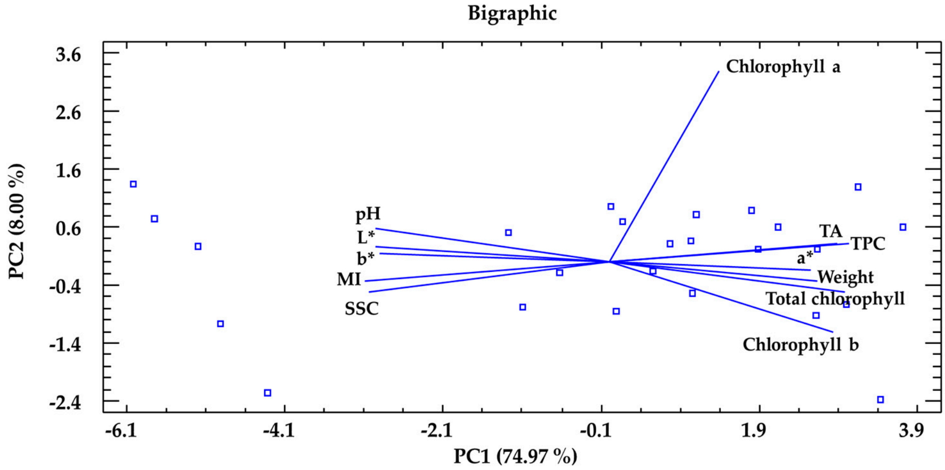

Supplementary Table S5a. This table shows the scores of the principal components

Row	*Label*	*Component 1*	*Component 2*	*Average Component 1*	*Average Component 2*
1	Broc_Control	3.70429	0.610437	**3.10**	**-0.23**
2	Broc_Control	2.60783	-0.926669		
3	Broc_Control	3.42372	-2.36199		
4	Broc_Control	2.62453	0.211814		
5	Broc_Control	3.15305	1.30035		
6	Broc_Tom	-6.01123	1.33619	**-5.24**	**-0.19**
7	Broc_Tom	-5.74895	0.734612		
8	Broc_Tom	-4.31414	-2.24397		
9	Broc_Tom	-4.91569	-1.05556		
10	Broc_Tom	-5.1909	0.272023		
11	Broc_Tom_$KMnO_4$	-1.26641	0.511595	**-0.56**	**0.24**
12	Broc_Tom_$KMnO_4$	-1.08945	-0.781102		
13	Broc_Tom_$KMnO_4$	0.0262665	0.963006		
14	Broc_Tom_$KMnO_4$	-0.621164	-0.196527		
15	Broc_Tom_$KMnO_4$	0.168601	0.688498		
16	Broc_Tom_$KMnO_4$_UV-C	1.10642	0.813546	**0.88**	**0.07**
17	Broc_Tom_$KMnO_4$_UV-C	0.0947379	-0.842801		
18	Broc_Tom_$KMnO_4$_UV-C	1.88204	0.223272		
19	Broc_Tom_$KMnO_4$_UV-C	0.546688	-0.165926		
20	Broc_Tom_$KMnO_4$_UV-C	0.777532	0.310271		
21	Broc_Tom_$KMnO_4$_UV-C_TiO_2	3.00495	-0.72326	**1.81**	**0.12**
22	Broc_Tom_$KMnO_4$_UV-C_TiO_2	2.14079	0.596155		
23	Broc_Tom_$KMnO_4$_UV-C_TiO_2	1.03713	0.36686		
24	Broc_Tom_$KMnO_4$_UV-C_TiO_2	1.80282	0.889211		
25	Broc_Tom_$KMnO_4$_UV-C_TiO_2	1.05652	-0.530034		

Supplementary Table S5b. ANOVA table for the component 1 scores according to the treatments

Source	*Sum of squares*	*FD*	*Mean Square*	*F-Ratio*	*P-Value*
Intergroup	207.007	4	51.7517	116.12	0.0000
Intragroup	8.91318	20	0.445659		
Total (Corr.)	215.92	24			

Supplementary Table S5c. Multiple comparisons test for the component 1 scores by treatments using Tukey HSD method

Treatments	*Replicates*	*Mean*	*Homogenous groups*
Broc_Tom	5	-5.23618	e
Broc_Tom_KMnO4	5	-0.556431	d
Broc_Tom_KMnO4_UVC	5	0.881484	c
Broc_Tom_KMnO4_UVC_TiO2	5	1.80844	b
Broc_Control	5	3.10268	a

Supplementary Table S5d. ANOVA table for the component 2 scores according to the treatments

Source	*Sum of squares*	*FD*	*Mean Square*	*F-Ratio*	*P-Value*
Intergroup	0.830705	4	0.207676	0.19	0.9424
Intragroup	22.2065	20	1.11033		
Total (Corr.)	23.0372	24			

Supplementary Table S5e. Multiple comparisons test for the component 2 scores by treatments using Tukey HSD method

Treatments	*Replicates*	*Mean*	*Homogenous groups*
Broc_Control	5	-0.233212	a
Broc_Tom	5	-0.191341	a
Broc_Tom_KMnO4_UVC	5	0.0676724	a
Broc_Tom_KMnO4_UVC_TiO2	5	0.119786	a
Broc_Tom_KMnO4	5	0.237094	a
